# A new audio cue to object weight resembles a naturalistic weight cue during movement planning but not during weight illusions

**DOI:** 10.1371/journal.pone.0325074

**Published:** 2025-06-02

**Authors:** James Negen, Heather Slater, Marko Nardini

**Affiliations:** 1 School of Psychology, Liverpool John Moores University, Liverpool, United Kingdom; 2 Psychology Department, Durham University, Durham, United Kingdom; University of Southampton, UNITED KINGDOM OF GREAT BRITAIN AND NORTHERN IRELAND

## Abstract

When a person picks up an object, naturalistic cues inform fine motor planning that is reflected in early spikes in force rate changes. Naturalistic cues to weight can also create an illusion whereby a signal to being heavier leads to the object being perceived as lighter – for example, the size-weight illusion. The present study asked to what extent an arbitrary new auditory cue, one that signals object weight, participates in these effects. In Experiment 1, participants used the new signal to adjust both their peak grip force rates and peak load force rates while lifting an object, consistent with using it for efficient motor planning. This matched how they used a naturalistic visual size cue. In Experiment 2, a new audio cue to heavier weight led to a heavier reported weight – the opposite of a size-weight illusion, and opposite to how the same participants used a naturalistic visual size cue. Thus, while the newly learned audio-weight mapping had similar functional properties to its more familiar perceptual counterpart, it did not show the same signature of automatic processing. These results have implications for understanding the flexible use of new cues and for targeting the underlying mechanisms in order to augment human abilities.

A sensory augmentation and substitution system (SASSy) is a device or technique that translates some information about the world into a different format that can be perceived by the user. For example, the EyeCane translates distances into tones or vibrotactile sensations [1], while the vOICe translates visual images into an auditory stream [2]. Devices such as these empower the user to understand some aspect of the world around them through a *new sensory skill*, a new mapping between sensory inputs and states of the world. This mapping might be a cognitive strategy, a perceptual phenomenon, or a mix – and could change in nature with practice. This study is part of a larger project aiming to understand if (how) such new sensory skills are used (un)like naturalistic perception. Here we first ask if participants can learn a new sensory skill that maps an arbitrary audio cue onto object weight to a point where it helps with motor planning, then go on to begin exploring if there is any evidence that such a mapping has any automatic qualities.

## Motor planning

To date, most tests of SSASys have focused on domains such as object identification [2,3] and navigation [1], but the potential for augmenting material perception – properties of objects, such as how heavy, slippery, or hot, they are – has been underexplored. To interact safely and efficiently with objects, we plan our movements based on naturalistic perceptual judgments of how heavy they will be [4–7]. Individuals with reduced vision, or those working in novel or hazardous environments, may make inaccurate judgments about object materials. Various disorders, in addition to typical aging, can make object handling less precise and more dangerous [8]. Mis-judgments are also possible in everyday settings, such as when we pick up an object we expected to be much lighter and have to adjust to its unexpected acceleration. The first goal of the present studies is to test to what extent a SASSy can help people efficiently adapt their movements to anticipate the weight of objects. We test this by measuring grip and load forces while participants pick up objects whose weight is signalled by a newly learned audio cue and/ or visual size (Experiment 1). This allows us to first compare the new sensory skill (the newly learned mapping between audio and weight) against a naturalistic weight cue in a very functional sense: a new sensory skill in the weight domain is generally more useful if it can help plan motor forces like naturalistic cues.

## Automatic processes

There are also ongoing debates over the extent to which a new sensory skill can become automatic [9–13]. We proceed here by adopting a conceptual analysis of automaticity from a previous article [10]. This analysis argues that automaticity includes a set of independent features. A process can be on a spectrum from less to more automatic depending on how many of these features it has and to what extent. Those are summarized as *unintentional*, *uncontrolled*/*uncontrollable*, *goal independent*, *autonomous*, *purely stimulus driven*, *unconscious*, *efficient*, and *fast* [10]. For example, an autonomous process is one that runs to completion with no need for conscious guidance or monitoring.

One of the proposed criteria for considering a process automatic is when its outputs ignore – or even run against – the use of explicit information [10]. The size-weight illusion is a striking example since people generally expect larger objects to be heavier, yet report the larger of two otherwise-similar objects as feeling lighter in the hand when the true weight is identical [14–18]. In this specific sense, the presence of an analogous illusion with a different cue would be a point in favour of viewing the use of that cue as more automatic. The second goal of the present studies is therefore to investigate whether a new auditory sensory skill for object weight exhibits this classic illusory interaction, leading people to report a lighter weight when we manipulate the audio cue to signal something heavier. We test this by training participants with a new audio cue to weight, presenting a manipulated lighter/ heavier signal, and comparing this against classic size-weight illusion trials (Experiment 2). This allows us to compare the new sensory skill (the newly learned mapping between audio and weight) against a naturalistic weight cue in terms of a key signature of automatic processing.

## Approach

Our studies followed standard methods for measuring ***Grip Force, Load Force, and Reported Perceived Weight*** during a ***Lifting Task.*** We briefly explain these measures here for readers who may not be familiar with them. Further details are given in the Methods as well as S1 File.

***Lifting task:*** An experimental task in which the participant is asked to lift objects repeatedly along a similar trajectory [4,14,19]. Tasks are designed to measure how motor control varies in response to sensory properties of the objects.

**Fig 1:**
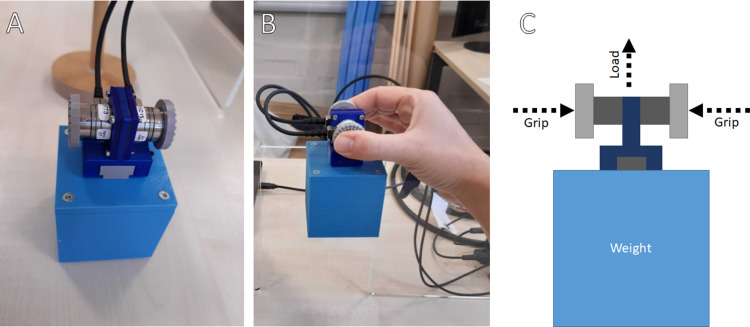
Equipment for a lifting task (A, B) and illustration of forces (C). Panel A shows the equipment from the same view as C. Panel B shows what the equipment looks like from the participant’s perspective during the lift (i.e., 90° rotated from A). The grip force is the ‘pinch’ used to keep the object from escaping. The load force is the ‘pull up’ used to lift the object.

***Grip force:*** The summed magnitude of forces normal to the gripping surface in Newtons. This is the ‘pinch in’ used to make sure an object does not escape during the lift (Fig 1). The rate at which this is changing is a *grip force rate* measured in Newtons per second (N/s). The maximum of this during a trial is the *peak grip force rate*, also Newtons per second – a measure that reflects planning as it usually occurs well before the object lifts [4]. A higher peak grip force rate indicates that the participant was prepared to lift a heavier object. This allows us to test if a new sensory skill can be used like naturalistic cues to anticipate the weight of objects and make appropriate motor planning adjustments.

***Load force:*** The summed magnitude of forces along the plane of the gripping surface. This is the ‘pull up’ used to lift an object (Fig 1). In the same way as above, the *peak load force rate* calculated from this reflects initial motor planning.

***Reported perceived weight:*** The subjective weight of an object as reported by a participant. Unlike the force rate measures, this is typically collected after the object has been held for some time, reflecting the full range of sensory inputs, feedback, and adjustment, and reflects an explicit judgment. There are often dissociations in reported perceived weight vs measured forces. For example, grip and load forces adapt almost immediately to the lifting of specific objects, while reported perceived weight does not particularly change without extensive experience and training [17,18].

## The present study

In Experiment 1, participants were asked to lift objects via a sensor array (Fig 1), planning their motor forces either by visual size alone or with the addition of a new audio cue to the object’s weight. This allowed us to test the hypothesis that a new signal for object weight will influence the forces used in the pre-planned stages of an object lifting task (the *motor planning adjustment hypothesis*), much like naturalistic weight cues. In Experiment 2, participants were asked to verbally estimate the weights of objects after lifting them, based on a new audio cue and/or holding it in their hand. This allowed us to test the hypothesis that use of a new signal for object weight will produce a “signal-weight” illusion analogous to the size-weight illusion (the *signal-weight illusion hypothesis*). Please see each experiment’s Introduction below for separate detailed reviews of related studies and the way the present experiments fit with them.

Before investigating any long-term training, we aim here to establish which effects can be seen early in learning. Our previous studies [20–23] suggest that new sensory skills can be learned and integrated with existing perception to at least some extent within an hour. In the present studies, similarly, we studied learning that took place over the course of about an hour.

## Experiment 1

This experiment was pre-registered here: https://osf.io/7gw2f. Experiment 1 tests to what extent a newly learned audio cue to weight and a more familiar visual size cue to weight influence peak grip force rates and peak load force rates during a lifting task. We already know that naturalistic cues to object weight can affect these outcomes. For example, participants readily calibrate the grip and load forces required to lift common objects on the first attempt [24], presumably drawing on a variety of cues such as size and material. While previous work already points towards the ability to learn new cues to weight that affect motor planning [25–30], the present study extends this by testing it in a setting that applies more directly to sensory augmentation (i.e., a novel counterbalanced mapping into the audio domain).

### Method overview

**Fig 2:**
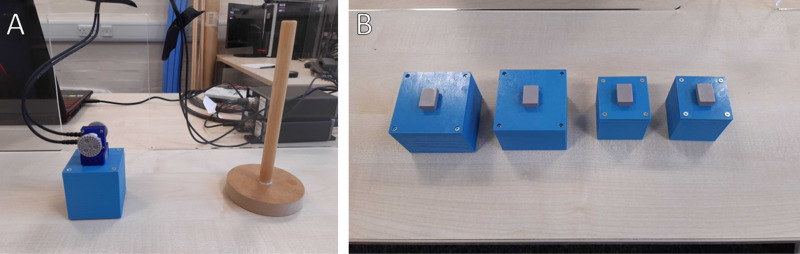
Apparatus for Experiment 1. Panel A shows a weight object with the sensor array attached and the reference stick. Panel B shows all four weight objects.

This experiment has a 2x2x2 full-factorial design. The factors are the object size (small or large), weight (lighter or heavier), and audio signalling (absent or present). Object size is designed to be only a partially reliable cue to object weight: a larger object is heavier on average than a smaller one, but there are two visually indistinguishable lighter and heavier versions of the smaller and larger object (thus, four objects in total – see Fig 2). In contrast, the audio signal is perfectly reliable – a different tone plays for each of the possible object weights. This design lets us check how participants use the familiar visual cue of size to plan picking up the object and whether, in the context of this cue not being perfectly reliable, they additionally begin to use the new audio cue to better distinguish the objects. If so, participants should respond to weight differences in the presence of the signal and differentiate their responses. In other words, we are looking for an interaction effect: the effect of weight on force measures will be larger when the signal is present than when it is absent. If found, this will provide evidence that a new audio cue to object weight can play a similar role as naturalistic weight cues in terms of planning motor forces.

### Relation to previous studies

We already know that various audio or visual signals, paired with different weights, can have some effect on the forces applied by the hand during a simple lifting task [25–30]. One early project paired a dark blue dot or single tone with 0.4 kg versus a bright red or double tone with 0.6 kg. The presentation of these colours/tones reduced hysteresis effects (e.g., too much force when presenting 0.4 kg just after 0.6 kg) to non-significance [25]. The next used a colour cue versus no cue versus a size cue; they also examined the effect of a simultaneous working memory task [28]. Mean forces tended to be higher/lower when presented with the corresponding colour. Further projects showed that either a probabilistic vertical cue like a traffic light (i.e., higher up means more likely to be heavy) or pictures of heavy objects can affect the forces used in the lift as well [29,30]. This all generally suggests that lifting forces can be affected without necessarily using the typical cues found in everyday experience.

Our approach builds on these previous studies with some design choices from a sensory augmentation perspective. First, rather than using single-direction mappings, we counterbalance these to ensure that “new” skills cannot be explained by systematic pre-existing biases or mappings. Second, rather than investigate issues such as linearity [29,30] and hysteresis [24], we more directly ask to what extent a newly learned cue has a reliable effect on determining grip and load force rates, indicative of efficient interactions with the object. Third, we assess the effects of new signals directly alongside a more familiar cue to weight: visual size. This allows us to put any effects in context and provides a pathway towards studies and settings in which familiar and new cues may be available at the same time (enhancing healthy perception, or augmenting perception that may be degraded, as with low vision, but still functional to a degree).

### Motor planning adjustment hypothesis

Experiment 1 tests the hypothesis that a newly learned sensory cue to object weight will influence the force rates used in the pre-planned stages of an object lifting task. More precisely: with peak load force rates or peak grip force rates as the outcome, there will be a signalling by weight interaction – the effect of weight on force rate measures will be larger when the signal is present than when it is absent, and with greater force rates applied to the heavier object. If confirmed, this would suggest that a new sensory skill rapidly takes on the role of aiding motor planning – similar to naturalistic cues to object weight. If not, it would suggest that either (a) a new sensory skill needs more time to begin aiding motor planning or (b) the domain of motor planning is relatively inflexible and only uses information sources that have been learned in development.

### Method

#### Participants.

Participant numbers were determined by a pre-registered stopping rule (see below). In the end there were 48 participants (18 male, 30 female; mean age of 21.4 years, standard deviation 3.6 years, minimum 18, maximum 35). Participants were recruited from the Durham, UK area. They were compensated with either £10 or with an hour of credit towards a system where people participate in each other’s studies. The procedure was approved by the Durham University Psychology Ethics Committee (Reference: PSYCH-2018-12-04). All experiments were performed in accordance with their guidelines and regulations. Written informed consent was obtained from all participants.

Because the expected effect size was difficult to establish, this experiment used a technique called the Pockock boundary [31] to set participant numbers. In this, specific criteria tests are pre-registered and then tested multiple times in a planned way that still controls the false positive rate. This done by pre-planning the exact outcome(s) that will lead to stopping, the exact points at which the data will be tested, and a lower acceptable p-value that will be taken as significant. In this case, we tested every 16 participants, up to 48, against a p-value of 0.0221. This procedure has a false positive rate of at most 5% yet allows for early stopping if the evidence of an effect is strong. In this experiment, the first two looks (16 and 32) did not meet the pre-registered stopping criteria and thus there were 48 participants. Note that because of this, we will still be testing against a p-value of 0.0221 throughout this experiment.

The final goal of 48 participants was found through simulation. Our simulations suggested, with this exact design and outcome of interest, that we should have 91% power to detect the interaction effect if it is at least 1/3 of a standard deviation. (Standard deviation referring to the unexplained noise.) This seemed to be a reasonable point of minimal theoretical interest; a smaller effect would say little about the scope to use this for sensory augmentation.

#### Apparatus.

The apparatus was designed to measure forces and torques placed on the objects (Figs 1 and 2). There was an upper assembly (a ‘clip’) that attached to each of the lifting objects on the top. This upper assembly had two force-torque transducers produced by ATI (Nano17, SI-25–0.25 standard). These were each capable of measuring forces and torques along all six axes with an error of at most 1.5% when the forces are up to 25 newtons and the torques up to 0.25 newtons. They each had a small flat pad, a grip surface, where the forces and torques were measured. These were mounted so that they are parallel to each other, rolled by 180°. A small piece of rough 3D printed material was mounted on the grip surface to increase grip. This upper assembly mounted to the top of the object in a way that made it possible and comfortable for the participant to grip it between the thumb and forefinger.

The upper assembly had two cables attached to it. These fed out signals that were then analysed by further equipment. The transducers plugged into interface power supplies provided by ATI. These were then fed to a data acquisition device (DAQ) made by National Instruments (NI USB6218). This DAQ recorded 1000 samples per second per channel. Data arrived into twelve channels, reported by USB to a laptop. The laptop used Windows and Matlab to process the data. It was transformed into force and torque measurements via a calibration matrix that was also provided by ATI.

The objects themselves were custom 3D printed boxes. There were four of them. The two sizes were small (7 cm on each edge) and large (10 cm). There was a small one that weighed 400g (small/lighter), a small one that weighed 600g (small/heavier), a large one that weighed 600g (large/lighter), and a large one that weighed 800g (large/heavier). Each pair of the same size was visually identical. The weight was achieved with an internal mixture of plasticine and 2 mm steel ball bearings such that weight was evenly distributed and did not shift when lifted. They each had a small mount on top to receive the upper assembly.

There was also a 21 cm tall stick (wooden dowel) that was used as a reference for how high to lift the object. While many previous studies have simply asked to participants to lift the object a small amount (e.g., “several cm” [32]), we used this visual reference in attempt to standardize the trials as much as possible. Since the sensors were already 10–13 cm above the table surface, participants were being asked to lift it 8–11 cm, which is very easy to achieve in a single swift motion.

#### Stimuli.

On every trial, the participant was able to see the object. On some trials, they also heard a sound indicating the weight. This was a pure tone that was 250ms in length. For half of participants, higher pitch indicated heavier weight (400 Hz to 400g, 1000 Hz to 600g, 2500 Hz to 800g). For the other half, lower pitch indicated heavier weight (2500 Hz to 400g, 1000 Hz to 600g, 400 Hz to 800g). Each tone had a ramp-up and ramp-down of 50ms where the amplitude increased/decreased linearly.

#### Procedure.

Participants were instructed beforehand that “Before lifting the object you will hear a sound which indicates that you can proceed. The sounds will be different on different trials and will be related to the weight of the objects, so you can use the sounds to help you tell how heavy the object will be.” On each trial, the participant was asked to close their eyes while the object was placed in front of them. Once placed, they were asked to open their eyes. If there was a sound on the trial, it played, the recording began, and the participant lifted the object. Otherwise, the experimenter began the recording and asked the participant to perform the lift. They were instructed to lift the object to the top of the reference stick, hold it briefly, and put it back down. Recording ended 5s after it began. The recording was shown to the experimenter without a labelled axis to prevent bias. The experimenter could re-do the trial if something had obviously gone wrong, e.g., the recording did not capture the full initial lift or the participant was already touching the sensors when the recording began.

The first 32 trials were a training block. These were not analysed; they were just done to build an association between weight and pitch for the participant. Each combination of small/large object and lighter/heavier weight was shown 8 times each in random order. All trials in this block featured the audio signal (which means they could not be subject to the main analysis anyway).

The next 32 trials were the testing block. These were analysed. Each combination of small/large, lighter/heavier, and with the audio signal present/absent, was each tested 4 times in random order.

#### Data processing.

See S1 File for full details. Raw data recordings were processed into peak load force rates and peak grip force rates. This was all done in the same way as previous research for comparison [4]. A specific trial was excluded as an outlier if it was more than 3 standard deviations from the mean of all other trials (including other participants) with the same parameters (small/large, light/heavy, signal present/absent). Means were taken within each participant and within repeated trials with the same parameters. In other words, each participant produced 16 final measures: every combination of small/large, lighter/heavier, signal present/absent (independent variables), and grip/load outcome (dependent variables).

#### Planned analysis.

As part of the Pocock procedure for potential early stopping, two outcomes were required to stop testing. With a pair of repeated-measures ANOVAs (separate for grip and load), we looked for a light/heavy by signal absent/present interaction in a specific direction. The factors were size (small/large), weight (lighter/heavier), signal (absent/present), and mapping, plus interactions. We specifically hypothesized (as pre-registered) that the effect of weight on force rate measures would be larger when the signal was present in a weight by signalling interaction. We hypothesized that this effect would also be in a specific direction, with force rates applied to lighter weights when signalled being smaller than force rates applied to heavier weights when signalled.

Please note that the coding of the weight variable is chosen here for the best ability to interpret the effect, which leads to a potentially counterintuitive choice. The weight variable refers to the object’s relative weight for its size (lighter/heavier), not the absolute weight (400/600/800g). This is because, with our coding here, the two sizes are evenly distributed across every combination of weight and signalling – all are tested with both the smaller and larger objects in exactly four test trials each. Any alternative coding would confound size with weight in the analysis.

### Results

#### Planned analysis.

**Fig 3:**
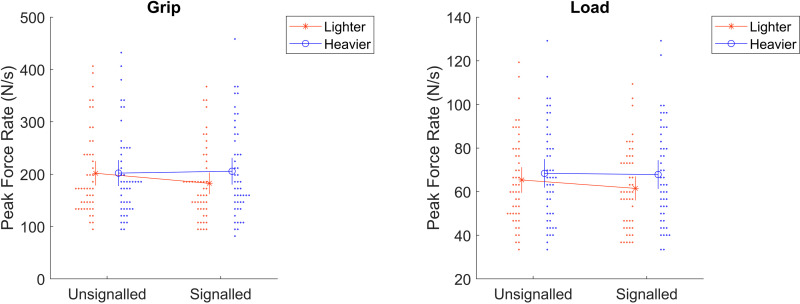
Means, 95% confidence intervals, and individual data points for the key interaction in the main hypothesis. Dots are individual participants that are stacked left/right in histogram bins for visual clarity.

Results are consistent with the motor planning adjustment hypothesis. The outcomes were not met after either 16 or 32 participants, so testing continued to 48 participants. The significance value equivalent to 5% was adjusted to 0.0221 as described above. 39 observations (1.27%) were excluded for being more than 3 standard deviations above the mean of all observations at the same size, weight, signal, and outcome (grip versus load). In a pair of 2 (size: smaller or larger) x 2 (weight: lighter or heavier) x 2 (signal: signalled or unsignalled) x 2 (mapping: higher lighter or higher heavier) mixed ANOVAs, the weight by signal interactions were both significant, F(1, 46) = 14.09, p < .001, η^2^ = .004 for grip, F(1, 46) = 6.16, p = .017, η^2 ^= .001 for load (full ANOVA results in S2 File). As Fig 3 shows, the effect of weight was larger when signalled than unsignalled, with a lower peak force rate for the lighter objects. In other words, peak force rates for lighter vs heavier objects were more differentiated when the signal was available, in the expected direction (greater force rates for heavier objects). This meets the criteria laid out in the pre-registration. This suggests that participants used the new sensory skill to aid their motor planning for how to grip and raise the object.

#### Additional results.

See S2 File for full details. In short, there were another eight significant findings (main effects of size, weight, signal, and mapping for each outcome). However, they do not speak for or against the interpretation given here. A main effect of size is likely due to the fact that the larger objects were on average heavier. The main effect of weight has the same explanation. The main effect of signal suggests that objects were treated as heavier when it was ambiguous. The main effect of mapping has no obvious interpretation and could be due to multiple comparisons. We also provide several more detailed displays/descriptions of the data, such as noting that the correlation between true weight and peak load force rate was higher for signalled trials than unsignalled trials.

### Discussion

The pre-registered hypotheses were confirmed, meeting the predictions of the motor planning adjustment hypothesis: the difference between heavier vs lighter weights was larger when given the signal; forces were higher when the object was heavier. In lay terms, the new audio cue helped people better calibrate and refine exactly how to plan the lift and apply the most appropriate force. Since a new sensory skill is defined as a new mapping between sensory inputs and states of the world, and since there was a fully counterbalanced mapping in the design here, we can be sure that these effects are due to the new sensory skill.

In terms of our broader theory, this points towards the new sensory skill being able to play a similar role to a naturalistic cue to object weight, at least in a very functional sense. This in turn suggest that there is good scope here for a SASSy to effectively augment perception – to be used to resolve ambiguity in object weight and help the user plan motor forces. It also suggests a level of immediate flexibility in this aspect of motor planning since the training was well under an hour.

This leads on to Experiment 2. The following experiment uses a comparable audio cue and training length. With the results of Experiment 1, we know that such a training regime already allows the new sensory skill to be useful in the sense of fine motor planning. This means that Experiment 2 can further examine the nature of this new sensory skill, particularly whether it shows a key marker of automatic processing: participation in weight illusions.

## Experiment 2

This experiment tests the hypothesis that use of a new signal for object weight will produce a “signal-weight” illusion analogous to the size-weight illusion (the signal-weight illusion hypothesis). This experiment was not pre-registered. However, the analysis was agreed before data collection as part of a student project.

With naturalistic signals to object weight, a signal that something is lighter leads to it being perceived as heavier when held [14]. The most famous is of course the size weight illusion: a smaller object feels heavier than a larger object of the same weight [14–18]. There is also the material weight illusion: an object that appears to be made of a less-dense material feels heavier than an object that appears to be made of a denser material of the same volume, despite identical weight [33,34]. There are also weight illusions related to darkness (light-coloured objects feel heavier) [35], shape (spheres vs cubes with large individual differences in direction) [36], and temperature (colder feels heavier) [37], which indicates that a broad scope of object properties can show these effects. While previous work already points towards at least some ability to learn weight illusions [38,39] – for example, golfers experience an illusion with practice golf balls that non-golfers do not experience [40] – the present study again extends this by testing in a way that applies more directly to sensory augmentation. This allows us to compare the new sensory skill against naturalistic weight cues in terms of a key marker of automatic processing.

### Method overview

The method, broadly summarized, is to have people (1) practice lifting visually identical cups with different weights and an audio pitch cue that matches the weight; (2) make sure they know the mapping by including trials where they hear the sound but do not touch the cup; and (3) test what happens if we give a lighter/heavier signal on matching objective weights to see if it resembles the size-weight illusion. This is done with half of participants given one mapping and half given the other. We used differently weighted opaque takeaway coffee cups, paralleling the naturalistic situation of lifting a cup with unknown contents. We also included trials with differently sized but equally weighted objects to test for the standard size-weight illusion within the same participants.

### Relation to previous studies

We already know that experience can induce certain weight illusions. For example, a female doll feels heavier than a male one [38] even when actual volume and weight are held constant. This seems like a case of learning through experience. Further, golfers perceive practice golf balls (which are typically lighter) as heavier than regular golf balls of equal weight [40], even though non-golfers perceive no weight difference. This also seems like it must necessarily be a result of specific experience. More directly, extensive training with a set of objects in which smaller objects are heavier leads to a complete reversal of the size-weight illusion [18]. In addition, expert echolocators experience a size-weight illusion after clicking to sense the size of the objects [41], which establishes at least some ability for new sensory skills in particular to generate weight illusions. All of this suggests there may be scope for training to lead to a new sensory skill becoming used for weight illusions in the same way as naturalistic cues like size or material, which would suggest that they are coming to be processed by similar automatic processes.

In contrast to previous studies, Experiment 2, like Experiment 1, is designed from a sensory augmentation perspective. We again use counterbalanced mappings, cancelling out any existing systematic biases to be sure that we are testing the effects of a *new* sensory skill. Second, we choose a simple signal (audio pitch) that could easily be employed in a sensory augmentation device. Third, by also including objects of different sizes, we directly compare any potential novel signal-weight illusion with a more standard size-weight illusion, serving as a control and to put performance with the new skill in context. This third point in particular contrasts the present study with previous work [41] where echolocation was used to sense size, generating a size-weight illusion rather than an independent route. As in Experiment 1, we also aim to establish which effects can be seen early in learning and use a short initial training timeframe of around 1 hour.

### Signal-weight illusion hypothesis

This suggests, as an analogue to the size-weight illusion, that the reported perceived weight will be lower when the new sensory skill signals that the object is heavier. More precisely: we hypothesize that the average natural logarithm of the ratio of reported perceived weights, specifically lighter divided by heavier, will be significantly more than zero. If confirmed, this will suggest that weight estimates via a new sensory skill rapidly come to participate in weight illusions similarly to those from naturalistic cues to object weight, suggesting that they are coming to be processed by similar automatic processes. If not, this could either suggest that (a) these processes are inflexible and ignore the new sensory skill when the object can be felt in the hand or (b) they use the new sensory skill in some way unlike naturalistic cues.

### Method

#### Participants.

There were 31 included participants (8 males, 24 females, 1 non-binary or prefer not to say; age mean of 23.67 years, standard deviation 6.14 years, minimum 18, maximum 42). Two more were excluded for the illusion analyses due to failure to learn the audio-weight mapping (30 years, female; 22 years, male – see *Planned Analyses* below for exclusion criteria). Participants were recruited from the Durham, UK area. They were compensated with either £10 or with an hour of credit towards a system allowing students to participate in each other’s studies. The procedure was approved by the Durham University Psychology Ethics Committee. All experiments were performed in accordance with their guidelines and regulations. Written informed consent was obtained from all participants.

Power was motivated by wanting, minimally, to show that the potential signal-weight illusion is either different from zero or different from the size-weight illusion. Power would therefore be the lowest if the signal-weight illusion was halfway between zero and the size-weight illusion. Since the size-weight illusion tends to be relatively large, d > 1 [17], this corresponds to approximately d = 0.5 or above. Given these considerations, the power given by this sample size is acceptable: 80% power to detect an effect of 0.5; 95% for 0.65; 99% for 0.77.

#### Apparatus and stimuli.

**Fig 4:**
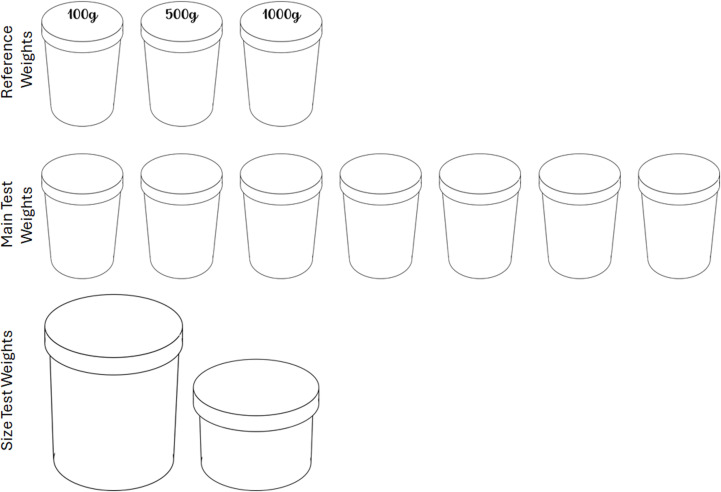
Objects used in Experiment 2. The reference weights were opaque coffee cups with their weight written on top. The main test weights were identical opaque coffee cups that ranged in weight from 200g to 800g. The size test weights were two plastic cylinders that both weighed 500g but were obviously different in size.

In addition to a standard Windows laptop with Matlab, several custom pieces were created. The first was a scale made with an Arduino, pressure sensors, and some 3D printed casing. The scale had internal software that would record the weight placed on it and report that measurement to the laptop. Reported weight was accurate within 2 grams for the range used here. In addition, there were three groups of weights: reference weights, main test weights, and size test weights (Fig 4). The reference weights and main test weights were made with identical opaque takeaway coffee cups and lids (130 mm tall, 90 mm diameter at top, 70 mm at base). The seven main test weights weighed 200, 300, 400, 500, 600, 700, and 800 grams. The three reference weights weighed 100, 500, and 1000g. Like Experiment 1, the weight was achieved with a mixture of plasticine and small steel ball bearings (essentially, a dense material that does not move inside or make noise when moved). The reference weights were only different from the test weights in the sense that their weight was written directly on top of the cup to be obvious and legible to the participant. The main test weights were visually identical to each other. The size test weights were two plastic cylinders, one larger (1L; 17.7 cm tall, 9.2 cm radius) and one smaller (0.35L; 8.2 cm tall, 8.5 cm radius). These items were sold commercially in a kitchen jar set. A sheet of construction paper was inserted to make these opaque. Both size test weights were weighted to 500g with the same mixture.

The audio cue to weight was generated in Matlab. The seven weights were designated by seven pure tones with a frequency from 150 to 1200 Hz, spaced linearly. Linear spacing in the auditory domain was chosen because the weights are also linearly spaced. The first half period was at 60% amplitude. The rest of the first half was at full amplitude. During the second half, the amplitude decayed at a rate of e^-4t, where t is the proportion of the second half that has passed. The stimuli each lasted 3 seconds. Participants were randomly assigned to either having these mapped with heavier corresponding to higher frequencies or heavier corresponding to lower frequencies.

#### Procedure.

**Fig 5:**
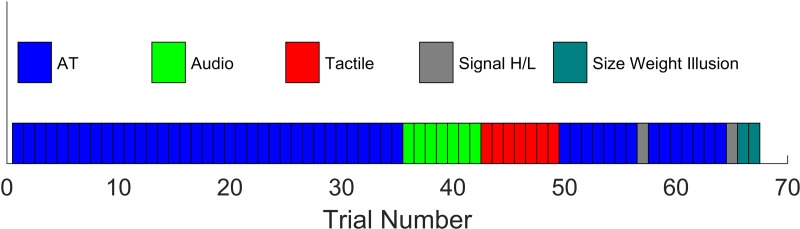
Quick reference for trial order. AT stands for audio-tactile. Signal H/L refers to either a Signal Heavier or Signal Lighter trial (order randomized).

The experiment lasted from about 30–60 minutes depending on the participant (Fig 5). There was first a short introduction before the trials. Participants were asked to feel how heavy the 100g, 500g, and 1000g reference weights are. They were introduced to the function of the scale, told that it weighs the things on it and sends a signal to the computer which plays different sounds to indicate the weight. They were also told that they will be asked to close their eyes so that they don’t see the weights moving between trials. The 100g and 1000g reference weight were removed. Participants were also told to report how heavy things “feel” to them as we were interested in their experience of the object’s weight (rather than their ability to anticipate an illusion and compensate for it).

The first 5 blocks of audio-tactile trials were designed to introduce the new sensory skill. On each trial, the participant closed their eyes while one of the seven main test weights was put on the scale. The participant then opened their eyes, lifted the 500g reference weight, put it down, waited for the sound, lifted the test weight, put it back down, and then gave a verbal numeric report of the perceived test weight. Each block was 7 trials long, using each of the 7 possible weights once in a random order (35 trials so far; see Fig 5). The trials could be answered based just on felt weight, but introduced participants to how the new sound was mapped to weight.

The remaining blocks then tested for various adaptations. Next was 1 block of audio-only trials (7 trials) to check that participants had learned the new mapping. These were the same as audio-tactile trial except they did not touch or lift the main test weight. Participants therefore had to judge weight (still using the reference standard as an anchor) using the tone, based on the mapping they had just been exposed to. Next was 1 block of tactile-only trials (7 trials). These were done to measure how accurate people were at the task without the new sensory skill. These were the same as the audio-tactile trials except without an audio stimulus. Next was another block of audio-tactile trials (7 trials) to refresh the audio training. The next trial (57th) was randomly either a signal heavy or signal light trial. This was done to test the signal-weight illusion hypothesis. Both were just like an audio-tactile trial from the perspective of the participant. Both used the 500g main test weight. A signal heavy trial played the audio stimulus that corresponded to 700g. A signal light trial played the audio stimulus that corresponded to 300g. Next was another block of audio-tactile trials (7 trials) to refresh training again. Next was another signal heavy/light trial (65th): if the 57th was signal heavy, this was signal light; if the 57th was signal light, it was signal heavy. This was also for testing the signal-weight illusion hypothesis.

To end, we gathered a size-weight baseline. These were the 2 size weight trials. In both, the participant was presented with a size test weight, asked to lift it, put it down, and then give a verbal report of its perceived weight. (The scale was not involved and there was no sound.) The smaller one was shown first.

#### Analysis plan.

For each participant, we calculated the correlation between the correct weight and the perceived weight on a logarithmic scale. These correlations were tested against zero with a t-test in order to check that participants had successfully learned the new mapping. After this, participants were excluded if their correlation was not statistically significant (i.e., r < 0.67, corresponding to p > .05). The signal-weight illusion was then calculated as a log-ratio, the natural logarithm of the signal lighter response divided by the signal heavier response. The size-weight illusion was a similar log-ratio, the natural logarithm of the smaller size response divided by the larger size response. These used natural logarithms so that the choice of numerator versus denominator does not affect the resulting variance. The final part of the plan then used t-tests to test the size-weight illusion against zero (the natural logarithm of one), the signal-weight illusion against zero, and the size-weight illusion against the signal-weight illusion.

### Results

#### Planned analyses.

Of the 33 participants, 31 showed a statistically significant correlation between the correct weight and the perceived weight on a logarithmic scale during audio-only trials. This was significantly above zero on average, t(32) = 13.73, p < 0.001, d = 2.39, including all 33. This confirms that the group learned the mappings they were trained with. The two participants with non-significant individual correlations (−.63, −.23) were then excluded from further analyses. The remaining 31 had a mean correlation of 0.88, median of 0.90, standard deviation of.10, and a range of 0.65 to 0.98.

**Fig 6:**
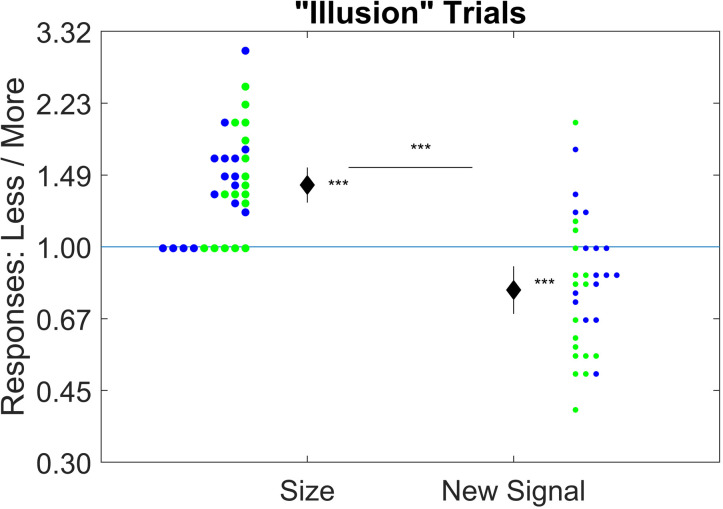
Results of Experiment 2. Graph shows means (diamond), 95% CI (bar through diamond), and histograms (dots to left/right) of the ratio of the two responses on “illusion” trials. Green dots are the heavier-higher mapping and blue are the heavier-lower mapping. For size, the smaller object’s judged weight is divided by the larger object’s judged weight. For the new signal, the lighter-signalled object’s judged weight is divided by the heavier-signalled object’s judged weight. The y-axis is on a logarithmic scale. *** significantly different from zero at the 0.001 level.

The classic size-weight illusion was replicated as the mean of the size-weight log-ratio was significantly above zero, t(30) = 7.19, p < 0.001, d = 1.29, mean 0.34 (Fig 6, left). This means that the smaller-sized object was reported as weighing about 40% more on average, despite their identical objective weight. In contrast, the potential signal-weight illusion was not found – instead, its opposite (Fig 6, right). The signal-weight log-ratio was significantly *below* zero, t(30) = −3.71, p < 0.001, d = −0.67, mean of −0.24. This means that the lighter-signalled weight was reported as weighing about 22% less on average, despite their identical objective weight. Unsurprisingly, there was also a significant difference between the size weight log-ratios and the signal-weight log-ratios, t(30) = 7.84, p < 0.001, d = 1.41. This is not in line with the signal-weight illusion hypothesis. Instead, it is consistent with averaging across or switching between the tactile signal and the audio signal.

#### Additional analyses.

See S2 File for full details. Briefly, the direction of the mapping did not have any particularly notable effect on outcomes of interest. Using weighted t-tests to compensate for the small difference in the number of included participants with each mapping did not affect results. No evidence for a dual-cue performance advantage was found. Comparing responses across trial types clarifies that participants did not just treat illusion trials like another tactile-only trial or another congruent audio-tactile trial with the same weight.

### Discussion

Although participants showed the standard Size-Weight illusion and learned the new mapping, the Signal-Weight Illusion hypothesis was rejected and we found significant evidence for its opposite. In other words, a larger object was reported as lighter; a new audio signal to an object being heavier lead to it being reported as heavier. This is interesting because it indicates that the new signal is influencing the reported weight, but in a manner different to the usual illusion.

The absence of a Signal-Weight illusion after the short training here suggests that, although the time was sufficient for people to make above-chance weight judgments with the new cue (in this experiment) and to efficiently adapt their lifting movements in response to it (in Experiment 1), it did not lead to the same kind of processing that leads to the classic Size-Weight illusion [14]. Since this phenomenon reflects relatively automatic processing, being resistant to explicit weight expectations [42], its absence limits the extent to which we can characterize the new sensory skill as automatic.

## General discussion

To summarize, one hypothesis was confirmed and one was rejected with a significant finding. In Experiment 1, the new pitch cue allowed people to adapt their motor planning to differentiate better between lighter versus heavier objects (i.e., larger gap between mean peak force rates). This confirms some scope for sensory augmentation in the domain of weight to contribute towards useful outcomes like efficient motor planning. On the other hand, in Experiment 2, while participants did adjust their reported perceived weights when given a new audio signal, they did so in the opposite direction of the size-weight illusion (i.e., lighter signal led to lighter estimates). This leads to a mixed answer to our core question of whether the new sensory skill can take on the same roles as naturalistic cues to object weight after short training; while the new sensory skill had similar functional properties to its more familiar perceptual counterpart (refining forces for movement), after short learning it did not show the hypothesized signature of becoming more automatic (weight illusion). Thus, the new sensory skill did show some functional similarity to familiar perceptual skills, but still appeared to depend on distinct underlying mechanisms.

On balance, the results suggest encouraging scope in terms of the potential use of a SASSy that helps with object weight. Results here show that participants can rapidly learn how one works and learn to adapt their planned motor forces. This experiment built on previous studies by showing that this is true even when the audio-weight mapping uses an arbitrary pitch cue and the mapping direction is counterbalanced in a way that cancels out pre-existing biases, confirming a high degree of flexible learning. This means that a well-designed SASSy for object weight could potentially be used by populations that have to especially rely on accurate motor planning or populations that have to operate in environments where naturalistic weight cues are particularly weak. In a broader sense, this may indicate even further scope for sensory augmentation approaches; if we frequently find that people can adapt well to new sensory skills, we may discover a wide variety of sensory augmentation opportunities that can be useful for any user (rather than targeted at a specific sensory issue).

Stepping back to see this result in context with others, we can observe that different situations create a spectrum of weight illusion effects that range from a strong contrast through to a strong enhancement. On the strong contrast end (signal to being lighter feels heavier) is the typical size-weight illusion. Recent work shows that this can be induced with echolocation in long-term expert users [41]. Moving away from the extreme, material cues also produce a contrast effect but usually smaller in magnitude [32]. At the midpoint of the spectrum, labelling objects as ‘light’ or ‘heavy’ does not appear to have any effect [43]. Moving into the enhancement end (signal to being heavier feels heavier), giving people information that makes a book sound more important makes it feel physically heavier [39]. The result here is also classed as an enhancement. One might also view the way that participants can be trained to experience a reversed size-weight illusion [18] as enhancement result since larger things are felt as heavier, though it may be better viewed as a contrast where the signal’s interpretation changes. It is not yet obvious how to predict where a new finding will lay along this contrast-enhancement spectrum. For example, one might think more basic object properties tend to produce a contrast and more high-level properties produce enhancement. However, that doesn’t explain why a doll perceived as physically stronger would be judged as lighter [38], independent of its perceived size. There could be some role of the kind of cue, the property being signalled, the extent of the experience, and a wide variety of other options.

### Limitations and future directions

The conclusions from the present studies are based on a very short period of training and experience. It is interesting that this was sufficient to incorporate a new signal into motor planning, but how results from explicit judgments and the opposite-to-predicted illusory effect might change with much longer training is now an open question.

The present study also does not particularly test an optimal model of how to plan grip/load forces. What we tested here is a directional hypothesis: higher weights leading to larger forces and the signal leading to a larger differentiation. This is fundamentally different from a model of the *best* forces (or force rates) to apply. For example, it is hard to say whether participants overshot and adjusted the forces by too much when they had the signal. It seems unlikely but there may be room to clarify this definitively if there is more work on optimal models of object lifting forces – this remains a challenge because of the biomechanical complexities of manual control, and the many potential ways to optimise movement (e.g., for different combinations of precision, speed, comfort, energy expenditure). Nevertheless, longer training studies should develop measures able to index changes in efficiency or precision of motor control as expertise is potentially gained over time.

One potential future direction is look for similar effects during more complex motor tasks. Lifting a small object straight up is a classic model system and a simple place to start. It is indeed something that people have to do in everyday life. However, it is not necessarily clear that similar new sensory skills would still be used in the same way if the task itself involved more complex or demanding motor outputs. It would help with generalization to potential applications to do further testing with more elaborate motor tasks.

Given the encouraging results here, it would be useful to look at further potential domains for augmentation as well. Replacements for vision have driven the majority of SASSy research but the results here show that people have some capacity to adapt in ways that do not particularly have to do with those efforts. It could be immensely useful to map out what kinds of domains are amenable to sensory substitution and augmentation.

## Conclusion

The project examined two ways that a new sensory skill to object weight might take on the same roles as naturalistic cues to object weight. We found that the new sensory skill can be used to adjust fine motor planning. However, we also found that the new sensory skill does not create the same weight illusions – rather, the ‘signal-weight illusion’ is in the opposite direction of things like the size-weight illusion or the material-weight illusion. This suggests that there is scope for sensory augmentation of object weight, at least in terms of fine motor behaviour. More generally, this could indicate significant potential scope for sensory augmentation techniques to be developed in additional domains. The findings also suggest that augmenting motor control is a separate outcome that can be achieved without becoming automatic in the same way as naturalistic cues. This has implications for understanding the flexible use of new cues and for targeting different underlying mechanisms in order to augment specific human abilities.

## Supporting information

S1 File Supplementary methods for Experiment 1. (DOCX)

S2 File Supplementary results for both experiments. (DOCX)
