# Supplementary material for: A new audio cue to object weight resembles a naturalistic weight cue during movement planning but not during weight illusions
Source: PLoS One. 2025 Jun 2;20(6):e0325074. doi: 10.1371/journal.pone.0325074 (PMC12129217; doi:10.1371/journal.pone.0325074)
Supplement: S1 File — (DOCX) [file pone.0325074.s001.docx]

Supplementary Methods For Experiment 1

This section details exactly how the transducer recordings were processed in Experiment 1. The raw recordings from each trial were a 5000x12 matrix of impedances through the force torque transducers (time points x channels; Figure 1-A). This required filtering and translating. After that, the required measures needed to be extracted. All of the choices here were consistent with a previous article that used the same sensor equipment and aimed to measure the same variables (Buckingham & Goodale, 2010). We detail this process below.


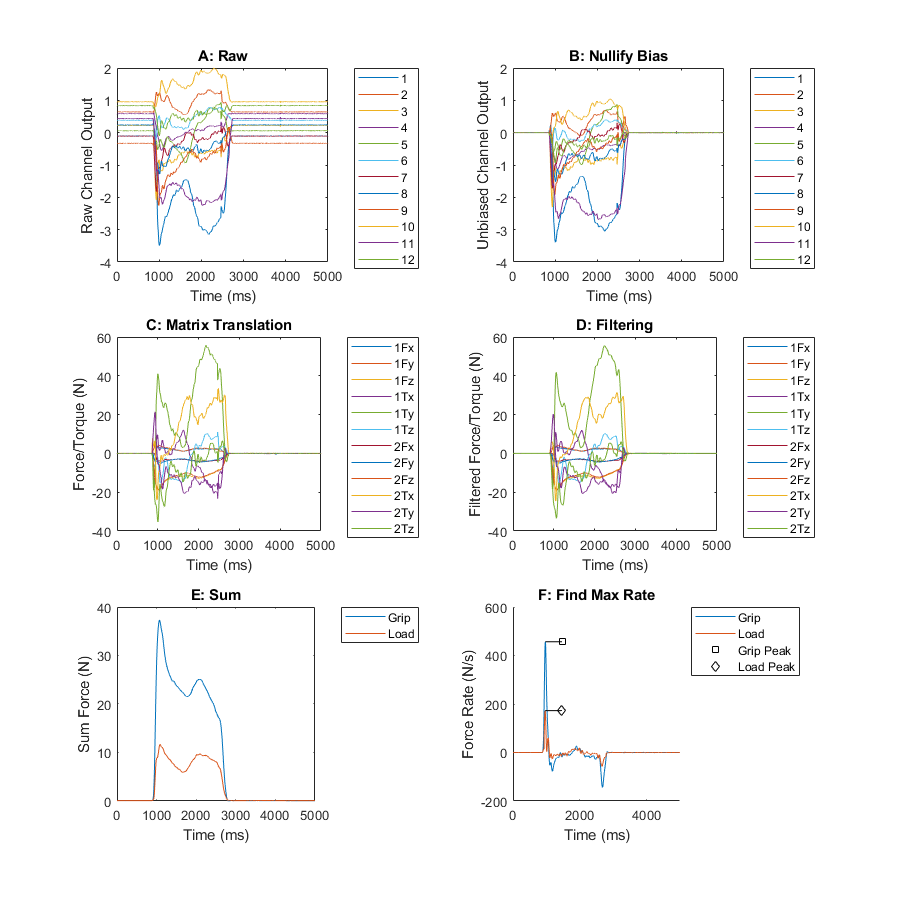


**Figure 1:** Example of how transducer signals are processed into peak grip force rates and peak load force rates.

Raw data were recorded in 12 channels (Figure 1-A). The first processing step was to nullify the bias for each channel (Figure 1-B). This was estimated as the mean of the first 64 samples in the same channel. This was subtracted from all measurements within the same channel. This was important because things like sensor temperature (which changes as they are handled by human hands) can alter the readings even when zero force is applied.

The second step was translation (Figure 1-C). Each transducer’s sample was matrix multiplied by the calibration matrix (1x6 sample times 6x6 matrix). These were then a set of forces and torques.

Next, each channel was then filtered twice through a fourth-order 14Hz Butterworth low-pass filter (Figure 1-D). The exact filter used here is somewhat arbitrary, although it is important that its response characteristics are flat in the lower range and provide a strong filtering effect for line noise (50Hz). 14Hz is a conventional cut-off where higher frequencies are thought unlikely to reflect motor planning (e.g. Buckingham et al., 2009; Buckingham & Goodale, 2010).

The grip force at each time-point was taken as the sum of the forces inwards, pushing the plates together (Figure 1-E). The grip force rate was calculated as the 5-point stencil of the grip force. The peak grip force rate was the maximum grip force rate during the trial (Figure 1-F).

The load force at each time point was found by first finding the total force tangential to the grip surface of each transducer (Figure 1-E). This was done by finding the magnitude of the vector sum of the two axes that each face along the grip surface (Pythagorean theorem). The total load force was the sum of this over the two transducers. This was also processed into a peak load force rate with a 5-point stencil and the maximum sample (Figure 1-F). These outputs then fed into the analysis as described in the main text.

The recording was also shown to the experimenter to review, though it was presented without a labelled axis to prevent bias. The experimenter could re-do the trial if something had obviously gone wrong e.g. the recording did not capture the full initial lift or the participant was already touching the sensors when the recording began. Curves were also checked after data collection for any remaining issues, though none were identified that were not already excluded by the automatic criteria (>3 SD from the mean of all trials with the same parameters).
