# Supplementary material for: A new audio cue to object weight resembles a naturalistic weight cue during movement planning but not during weight illusions
Source: PLoS One. 2025 Jun 2;20(6):e0325074. doi: 10.1371/journal.pone.0325074 (PMC12129217; doi:10.1371/journal.pone.0325074)
Supplement: S2 File — (DOCX) [file pone.0325074.s002.docx]

Supplementary Results for Both Experiments

**Experiment 1 Additional Results**

In Experiment 1, there were eight significant findings that resulted from the pre-registered analysis that are not particularly part of the hypothesis (Figure 1; Tables 1-2). Six are side-effects of the main hypothesis or the experiment design. These are significant main effects of size, weight, and signalling for both outcomes. Participants were able to see the difference in size and the larger objects did in fact weigh more on average. This explains the size main effects (greater for larger). Participants had the signal to show them that the heavier objects were heavier on half of trials. This explains the weight main effects (greater for heavier). When participants were not given a signal, the forces they applied were similar to the heavy signalled trials (Figure 1). In other words, without information, they erred on the side of the objects being heavier. This creates the signalling main effects (greater for unsignalled): all unsignalled trials are treated as heavy, whereas half of signalled trials are treated as light. While these effects are not vital to the main hypothesis and were not part of the pre-registered criteria, they do fit with it.

Two additional significant findings do not have an obvious interpretation. Participants created greater force rates with the higher lighter mapping than the higher heavier mapping, F(1, 46) = 8.51, p = .005, η^2^ = 0.129 for grip; F(1, 46) = 7.88, p = .007, η^2^ = 0.122 for load. The two are likely related as greater load forces require the use of greater grip forces to keep the item from escaping. It is not obvious why this happened but it may be a simple Type 1 error. The analysis for this experiment involved calculating 30 p-values and in that sense, it is not surprising to see a low p-value without a theoretical explanation. We also note that while mapping did have a significant main effect, it did not significantly interact with any other effect at the level required by the Pocock procedure here (.0221). To give this result in full, Figure 1 shows the complete breakdown of data into all possible categories: mapping (circles versus asterisks), weight, size, signalling, and grip versus load.


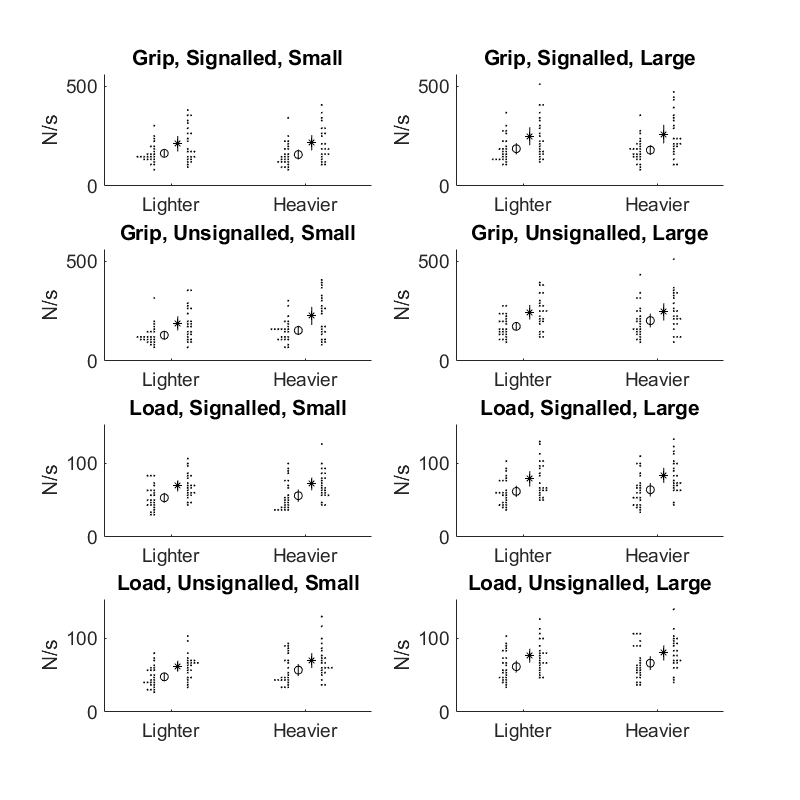


**Figure 1:** Full breakdown of outcome measures. Individual dots are participants. Circles are for the higher heavier mapping mean. Asterisks are for the higher lighter mapping mean. Error bars are 95% confidence intervals.

Table 1: Within-Subjects Effects on Peak Grip Force Rates.

|  | df | F | p | η^2^ |
| --- | --- | --- | --- | --- |
| **Size** | **1** | **81.2889** | **< .001** | **0.046** |
| Size ✻ Mapping | 1 | 0.3023 | 0.585 | <0.001 |
| Residual | 46 |  |  |  |
|  |  |  |  |  |
| **Weight** | **1** | **12.3055** | **0.001** | **0.005** |
| Weight ✻ Mapping | 1 | 0.2787 | 0.600 | <0.001 |
| Residual | 46 |  |  |  |
|  |  |  |  |  |
| **Signalling** | **1** | **6.1135** | **0.017** | **0.002** |
| Signalling ✻ Mapping | 1 | 0.0182 | 0.893 | 0.000 |
| Residual | 46 |  |  |  |
|  |  |  |  |  |
| Size ✻ Weight | 1 | 1.0347 | 0.314 | <0.001 |
| Size ✻ Weight ✻ Mapping | 1 | 1.8735 | 0.178 | 0.001 |
| Residual | 46 |  |  |  |
|  |  |  |  |  |
| Size ✻ Signalling | 1 | 3.4899 | 0.068 | 0.001 |
| Size ✻ Signalling ✻ Mapping | 1 | 4.8971 | 0.032 | 0.001 |
| Residual | 46 |  |  |  |
|  |  |  |  |  |
| **Weight ✻ Signalling** | **1** | **14.0941** | **< .001** | **0.004** |
| Weight ✻ Signalling ✻ Mapping | 1 | 3.1788 | 0.081 | 0.001 |
| Residual | 46 |  |  |  |
|  |  |  |  |  |
| Size ✻ Weight ✻ Signalling | 1 | 3.1787 | 0.081 | 0.001 |
| Size ✻ Weight ✻ Signalling ✻ Mapping | 1 | 5.4631 | 0.024 | 0.001 |
| Residual | 46 |  |  |  |

*Note*: Significant effects in bold face. p < .0221 required for significance.

Table 2: Within-Subjects Effects on Peak Load Force Rates.

|  | df | F | p | η^2^ |
| --- | --- | --- | --- | --- |
| **Size** | **1** | **152.78600** | **< .001** | **0.058** |
| Size ✻ Mapping | 1 | 0.73129 | 0.397 | <0.001 |
| Residual | 46 |  |  |  |
|  |  |  |  |  |
| **Weight** | **1** | **32.11581** | **< .001** | **0.011** |
| Weight ✻ Mapping | 1 | 0.00502 | 0.944 | <0.001 |
| Residual | 46 |  |  |  |
|  |  |  |  |  |
| **Signalling** | **1** | **6.84024** | **0.012** | **0.003** |
| Signalling ✻ Mapping | 1 | 3.26918 | 0.077 | 0.001 |
| Residual | 46 |  |  |  |
|  |  |  |  |  |
| Size ✻ Weight | 1 | 1.97960 | 0.166 | <0.001 |
| Size ✻ Weight ✻ Mapping | 1 | 0.41010 | 0.525 | <0.001 |
| Residual | 46 |  |  |  |
|  |  |  |  |  |
| Size ✻ Signalling | 1 | 3.54279 | 0.066 | 0.001 |
| Size ✻ Signalling ✻ Mapping | 1 | 0.35636 | 0.553 | <0.001 |
| Residual | 46 |  |  |  |
|  |  |  |  |  |
| **Weight ✻ Signalling** | **1** | **6.15927** | **0.017** | **0.001** |
| Weight ✻ Signalling ✻ Mapping | 1 | 0.78649 | 0.380 | <0.001 |
| Residual | 46 |  |  |  |
|  |  |  |  |  |
| Size ✻ Weight ✻ Signalling | 1 | 2.93852 | 0.093 | 0.001 |
| Size ✻ Weight ✻ Signalling ✻ Mapping | 1 | 0.14242 | 0.708 | <0.001 |
| Residual | 46 |  |  |  |

Another view of these data may be helpful. Examination of correlations suggests that the presence of the signal refined motor planning as reflected by increased correlation between true weights and peak forces. When unsignalled, the correlation was 0.38 and 0.56 for grip and load. When signalled, these increased to 0.57 and 0.65. In this sense, the new sensory signal did not merely bias or replace the naturalistic size cue, which was also present although not fully reliable. In context, the presence of the signal led to peak force rates that were more tightly correlated with the true weight.

We also provide a view into the change in peak force rates over the course of the experiment. Figure 2 shows the mean peak force rates for every type of signalled trial, normalized to each participant (i.e. with the participant mean subtracted), broken down by how many times the participants had experienced that specific condition (including training). Objects with the same size but a different signalled weight (blue vs red) were treated in very similar ways at the start, and as late as the 8^th^-9^th^ exposures. By the final exposure, however, the peak load force rates were nearly the same for the small heavy and large light objects (which did in fact both weigh 600g) and the peak grip force rates had a stable order with heavier over lighter.

**Figure 2**: The Signal by Weight Interaction Developing over the Experiment. Only signalled trials are included here. Over time, the peak force rates became better differentiated by the signalled object weight (blue versus red). Error bars are 95% confidence intervals. The Y axis is normalized by subtracting the participant mean.

**Experiment 2 Additional Results**

To begin, we looked at the two mappings separately. For participants who had the heavier is higher mapping, the log-ratio was significantly below zero, t(16) = -3.29, p = 0.005, d = -0.80. For participants who had the heavier is lower mapping, the log-ratio was not significantly different from zero, t(13) = -1.85, p = 0.088, d = -0.49. The difference between the two groups was not significant, t(29) = -1.34, p = 0.191, d = -0.48. In other words, we did not find any particularly strong reason to think that there was a meaningful difference between the two mappings.

We also explored what happens if weightings are used to correct the slight difference in the number of included participants with each mapping (17 vs 14). The signal-weight illusion remains significant and in the same direction when giving each participant in the over-represented mapping a weighting of 14/17, t(27) = -3.72, p < 0.001, d = -0.70, mean of -0.23. In other words, the signal-weight illusion here does not appear to be an artifact of uneven mapping assignment.

Further, performance among the three trial types was also compared. The average log-error was 0.40 for audio-only trials, 0.26 for tactile-only trials, and 0.28 for audio-tactile trials. This suggests that audio-tactile trials were not particularly more accurate than tactile-only at this point in training. Further study would be required to see if explicit judgements are made more accurate with an audio cue after a longer training period.

Finally, two additional analyses were done to be sure that the responses on the signal light and signal heavy trials are statistically separable from audio-tactile / tactile-only trials with the same weight. First, a one-way repeated-measures ANOVA was done with the signal light trial, signal-heavy trial, and the final 500g audio-tactile trial before these illusion trials. The Greenhouse-Geisser correction was used. There was a significant effect of trial type, F(1.60, 47.94) = 11.3, p < .001. This suggests that the illusion trials are treated differently from congruent audio-tactile trials with the same weight. A similar analysis compared the signal light trials, signal heavy trials, and the tactile-only 500g trial. This also showed a main effect of trial type, F(1.93, 57.82) = 8.16, p < .001. This suggests that the illusion trials are treated differently from tactile-only trials with the same weight. For both analyses, the signal light mean is the lowest and the signal heavy mean is the highest. This in turn reinforces the conclusion that there is a signal weight effect (though in the opposite direction) since it directly shows that the illusion trials were not treated the same way as audio-tactile or tactile-only trials with the same weight.
